# Locomotor activity in *Aedes aegypti* with different insecticide resistance profiles

**DOI:** 10.11606/s1518-8787.2021055002809

**Published:** 2021-04-08

**Authors:** Bruno Magalhães Nakazato, Maria de Lourdes da Graça Macoris, Paulo Roberto Urbinatti, Tamara Nunes Lima-Camara

**Affiliations:** I Universidade de São Paulo Programa de Pós-Graduação em Saúde Pública Faculdade de Saúde Pública São PauloSP Brasil Universidade de São Paulo. Faculdade de Saúde Pública. Programa de Pós-Graduação em Saúde Pública. São Paulo, SP, Brasil; II Superintendência de Controle de Endemias Laboratório de Entomologia Aplicada MaríliaSP Brasil Superintendência de Controle de Endemias. Laboratório de Entomologia Aplicada. Marília, SP, Brasil; III Universidade de São Paulo Faculdade de Saúde Pública Departamento de Epidemiologia São PauloSP Brasil Universidade de São Paulo. Faculdade de Saúde Pública. Departamento de Epidemiologia. São Paulo, SP, Brasil

**Keywords:** *Aedes*, Insecticide Resistance, Locomotion, Disease Vectors

## Abstract

**OBJECTIVE:**

To evaluate locomotor activity in four field populations of *Ae. aegypti* with different insecticide resistance profiles from the state of São Paulo for two years.

**METHODS:**

This study comprised the susceptible Rockefeller strain and four populations from São Paulo, Brazil: two considered populations with “reduced susceptibility” to pyrethroids (Campinas and Marília), and two “resistant populations” (Santos and Ribeirão Preto). First, 2016 and 2017 eggs from these five populations were hatched in laboratory. Virgin females underwent experiments under laboratory conditions at 25°C, with 12:12h light/dark (LD) photoperiod; 24-hour individual activity was recorded using a locomotor activity monitor (LAM).

**RESULTS:**

In females from 2016 field populations, both resistant populations showed significant more locomotor activity than the two reduced susceptibility populations and the Rockefeller strain (p < 0.05). As for females from 2017 field populations, reduced susceptibility populations showed a significant increased locomotor activity than the Rockefeller strain, but no significant difference when compared to Santos resistant population (p > 0.05).

**CONCLUSIONS:**

Our results indicate that insecticide-resistant *Ae. aegypti* populations show increased locomotor activity, which may affect the transmission dynamics of their arboviruses.

## INTRODUCTION

*Aedes* (*Stegomyia*) *aegypti* (Linnaeus, 1762) is a mosquito highly adapted to the urban environment, often found inside human dwellings and their surroundings^1^^–^^3^. This diurnal and anthropophilic species is the primary vector of several arboviruses, such as dengue, chikungunya, and Zika – all mainly transmitted to humans through the bite of an infected female *Ae. aegypti*^3^.

Among the measures adopted to control *Ae. aegypti*, using chemical insecticides has been proposed as chemical control, eliminating or relocating breeding sites as mechanical control and applying *Bacillus thuringiensis insraelensis (Bti*) larvicide as biological control^4^. With dichloro-diphenyl trichloroethane (DDT) development in 1939 new prospects loomed regarding the species, including its eradication in Brazil and neighboring countries by 1958^5^^–^^7^. In general, all chemical insecticide classes act on mosquito's central nervous system (CNS), inducing the “knockdown effect”^8^.

Besides the organochlorine class (OC), which contains DDT, chemical organophosphate (OP), carbamate (CA), and pyrethroid (PY) insecticides are also used against *Ae. aegypti* and other mosquito vectors^8^^,^^9^. Whereas OC and PY act by destabilizing the balance and passage of sodium and potassium ions though the voltage-gated sodium channel (NaV)^10^, OP and CA act by phosphorylating acetylcholinesterase (AChE), preventing acetylcholine (Acetyl-COA) degradation and thus disrupting the following neuron action potential^8^.

The massive use of chemical insecticides may lead different *Ae. aegypti* populations to develop resistance. The first case of DDT-resistance in *Ae. aegypti* populations was reported in Cúcuta, Colombia, in 1957^7^. Besides resistance, DDT also incur a long residual action in the environment, affecting agriculture and aquatic ecosystems, besides having a hazardous effect on human health, so that its use in the Americas declined in the early 1970s^9^^,^^11^.

In Brazilian *Ae. aegypti* populations, insecticide resistance was first observed in 1994, with reduced susceptibility to organophosphates^12^. Due to *Ae. aegypti* decreased sensitivity to PY and OP, several populations of this mosquito have been monitored in São Paulo^12^, indicating high levels of PY resistance, especially in the cities of Santos and Ribeirão Preto^13^^,^^14^, highly urbanized areas. In turn, *Ae. aegypti* populations from Marília and Campinas showed low levels of PY-resistance and susceptibility to OP insecticides^13^^,^^14^.

The World Health Organization (WHO) classifies mosquitoes susceptibility profile according to mortality, as follows: > 98% mortality is deemed susceptible; < 98% as suspected resistance; 90–97% as suspected presence of resistant genes; and < 90% as confirmed presence of resistant genes^15^. Mosquito vectors acquire resistance to PY through the structural alteration of specific genes due to random and non-synonymous substitutions, encoding altered target proteins and reducing insecticides molecules binding^16^. Such mutations, known as “knockdown resistance” (*Kdr)*, were first detected in *Musca domestica* as a leucine-to-phenylalanine substitution (*L*1014*F*) and are passed down to new generations^16^^,^^17^. Two mutations are widespread among Brazilian *Ae. aegypti* populations: phenylalanine-to-cysteine (*F*1534*C*) and valine-to-isoleucine (*V*1016*I*) substitution – both related to the phenotypic profile of pyrethroid resistance^14^^,^^18^^,^^19^.

The daily activities of mosquito vectors, such as mating, blood-feeding, flight, and oviposition, are controlled by a circadian clock^20^. *Ae. aegypti* often presents a diurnal and bimodal locomotor activity, with morning and afternoon peaks^2^. However, insemination, blood-feeding^21^, and dengue infection^22^ may influence this species’ locomotor activity and consequently its arbovirus transmission dynamics^23^. Brito et al. (2013) found *Ae. aegypti* with *Kdr* mutations to show a significant increase in locomotor activity when compared to the Rockefeller strain^24^. Considering that, studies evaluating the locomotor activity of resistant populations are necessary for providing a better understanding of the effects of insecticide resistance on *Ae. aegypti* locomotor activity.

We assess the hypothesis that insecticide-resistant *Ae. aegypti* populations present an increased locomotor activity, potentially favoring arboviruses transmission dynamics. This study sought to assess locomotor activity of four *Ae. aegypti* populations with different insecticide resistance profiles from the state of São Paulo over two years.

## METHODS

### Population Profiles

**Figure 1:**
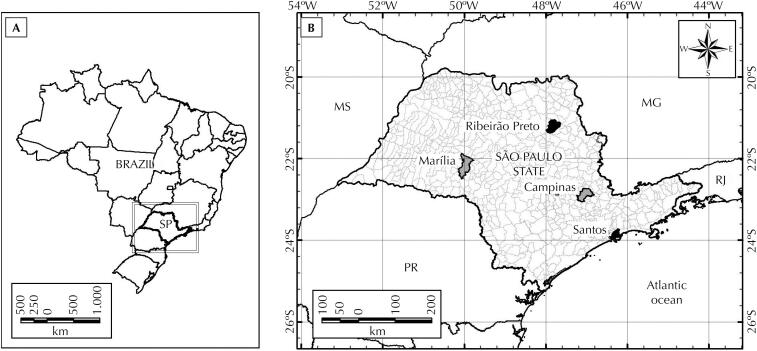
*Ae. aegypti* population profile according to insecticide resistance. (A) Brazil Map highlighting the state of São Paulo. (B) São Paulo municipalities with the *Ae. aegypti* populations tested. Reduced susceptibility to pyrethroids (grey – Campinas and Marília) and resistant (black – Santos and Ribeirão Preto).

*Aedes aegypti* populations from São Paulo, Brazil, were selected by the phenotype profile of insecticide resistance. Based on bioassays tests^14^ and following WHO criteria^15^, populations from the municipalities of Campinas (22°54’26’’S; 47°3’48’’W) and Marília (22°12’50’’S; 49°56’45’’W) were considered as presenting “reduced susceptibility” to pyrethroid (PY), whereas populations from Santos (23°56’41’’S; 46°19’49’’W) and Ribeirão Preto (21°10’39’’S; 47°48’37’’W) were considered “resistant” (Figure 1). We also tested the Rockefeller strain (from the Centers for Disease Control and Prevention-CDC, San Juan, Puerto Rico), a laboratory reference strain susceptible to insecticides and frequently used for monitoring *Ae. aegypti* field populations biological responses^13^. For presenting a > 98% mortality to pyrethroids (susceptible), this strain enables the comparison with reduced-susceptibility and resistant populations. *Ae. aegypti* populations from 2016 and 2017 underwent experiments, except for Ribeirão Preto, which was only tested in 2016. Eggs were collected using ovitraps during Spring – the pre-epidemic season. The susceptibility/resistance status of non-blood-fed female *Ae. aegypti* (2–5 days old) was evaluated based on mortality rates obtained from bioassays using insecticide-impregnated papers, according to WHO methodology^14^^,^^15^. Insecticide dose was established as the double of 99% lethal dose obtained to a susceptible strain.

### Mosquito Rearing

For hatching, eggs from each population were placed in 10 × 10 × 5 cm labeled trays containing 400 mL of tap water and 0.2 mg of fish food (Tetramin®). Larvae were kept in 30 × 20 × 5 cm labeled trays with 1 L of tap water and 20 mg of fish food. Hatching trays were kept in an incubator at 25° Celsius and 70% relative humidity with 12 hours of light and 12 hours of dark (LD 12:12). All pupae were placed in individual small containers with 5 mL of tap water until emergence, ensuring virgin females. Then, females from each population were transferred to labeled cages and kept in the same incubator under the same photoperiod, temperature, and humidity as the rearing phase.

### Analysis of Locomotor Activity

Three-to-four-days-old females from all tested populations (Campinas, Marília, Santos, Ribeirão Preto and Rockefeller) were anesthetized in ice and then placed in individual glass tubes (1 cm x 10 cm). Adults were fed by a 10% sucrose-soaked cotton plug. Glass tubes were sealed with Parafilm® and placed in the locomotor activity monitor (TriKinects Inc, Waltham, MA, USA), which was equipped with infrared beam and detectors, counting every time mosquitoes broke the beam and measuring their activity-rest pattern^25^^,^^26^. All monitors were placed within a Precision Scientific Incubator Model 818, under LD 12:12 and constant temperature of 25ºC. In our experiments, light functioned as the *Zeitgeber* (ZT) – a Germanic term that means “time giver” or synchronizer^21^. In a LD 12:12 cycle, for example, ZT02 corresponds to 2 hours after lights are on and ZT14 to 2 hours after lights are off.

Each female's total activity was individually recorded for seven consecutive days, at 30-minute intervals, using the DAMSystem data acquisition software. At least two replicate experiments were conducted for all populations in 2016 and 2017 (except for Ribeirão Preto, which only comprised populations from 2016). Only females that survived until the sixth day of the experiment were analyzed (Table 1), and data from the second to the fifth day of the experiment were used for analysis.

**Table 1:** Means of female *Ae. aegypti* locomotor activity according to insecticide resistance profile.

Year	Profile	Population	n	Mean (SD)		
				Total activity (24h)	Diurnal activity without lights-on (11.5h)	HDA (ZT06 to ZT12)
2016	Reduced	Campinas	95^b^	0.43 (0.16)	0.68 (0.26)	0.83 (0.28)
	susceptibility	Marília	104^b^	0.43 (0.19)	0.62 (0.25)	0.79 (0.26)
	Resistant	Santos	68^a^	0.48 (0.17)	0.73 (0.25)	0.91 (0.28)
		Ribeirão Preto	73^a^	0.57 (0.21)	0.83 (0.30)	0.97 (0.29)
	Susceptible	Rockefeller	75^a^	0.45 (0.16)	0.62 (0.21)	0.78 (0.23)
2017	Reduced	Campinas	96^b^	0.58 (0.20)	0.83 (0.31)	0.96 (0.33)
	susceptibility	Marília	43^a^	0.46 (0.18)	0.74 (0.29)	0.91 (0.31)
	Resistant	Santos	63^a^	0.56 (0.20)	0.86 (0.32)	1.00 (0.35)
	Susceptible	Rockefeller	72^a^	0.45 (0.16)	0.58 (0.22)	0.73 (0.24)

n: total number of individuals tested; SD: standard deviation; HDA: half-day activity.

a Two replicates.

b Three replicates.

William's mean (Wm) was calculated as an estimate of the central tendency activity during each 30 minute-interval^21^^,^^16^. William's mean is a modification of geometric mean related to data log-transformation to accommodate zero values by adding 1 to all data values and then subtracting 1 from the mean^27^^,^^28^.

### Statistical Analysis

The means of total activity (24 hours), diurnal activity without lights-on (excluding the first 30 minutes of the diurnal phase lights-on due to the “startle response effect” resulting from the dark-light transition)^21^, and half-day activity (HDA; ZT06 to ZT12 interval) were calculated for each mosquito. Each mosquito mean HDA, calculated using all log (N+1) values related to every ZT06-ZT12 30-min interval, was employed in statistical analyses. We chose half-day activity for statistical analyses because *Ae. aegypti* shows greater activity during this time of the day^2^^,^^29^. Previous studies have also used this method to calculate the total, diurnal, and nocturnal *Ae. aegypti* locomotor activity means for statistical analysis^21^^,^^27^.

After calculating mean HDA, the Kolmogorov-Smirnov test was use to assess data normality. Once normality assumptions were satisfied, the parametric independent samples T-test was used to verify differences between replicas of each population in each year (when three replicas were present, one-way ANOVA was used). Independent samples T-test was also used to compare possible differences within the same population between years (temporal variation). One-way ANOVA and Fisher's LSD *post-hoc* test compared locomotor activity between populations for the two years evaluated and among all five tested populations. Populations with reduced susceptibility or with resistant profile were not grouped for statistical analyses. All statistical analyses were performed in the software Statistical Package for the Social Science (SPSS), version 17, with α < 0.05.

## RESULTS

We assessed 542 virgin females from four *Ae. aegypti* field populations (resistant and susceptible) and 147 the Rockefeller strain (Table 1). Table 1 shows the number of females within each tested population for 2016 and 2017 and the means of total activity, diurnal activity without lights-on, and half-day activity (HDA – ZT06 to ZT12 interval). All populations presented higher means in the ZT06-ZT12 interval, when *Ae. aegypti* showed more locomotor activity.

**Figure 2:**
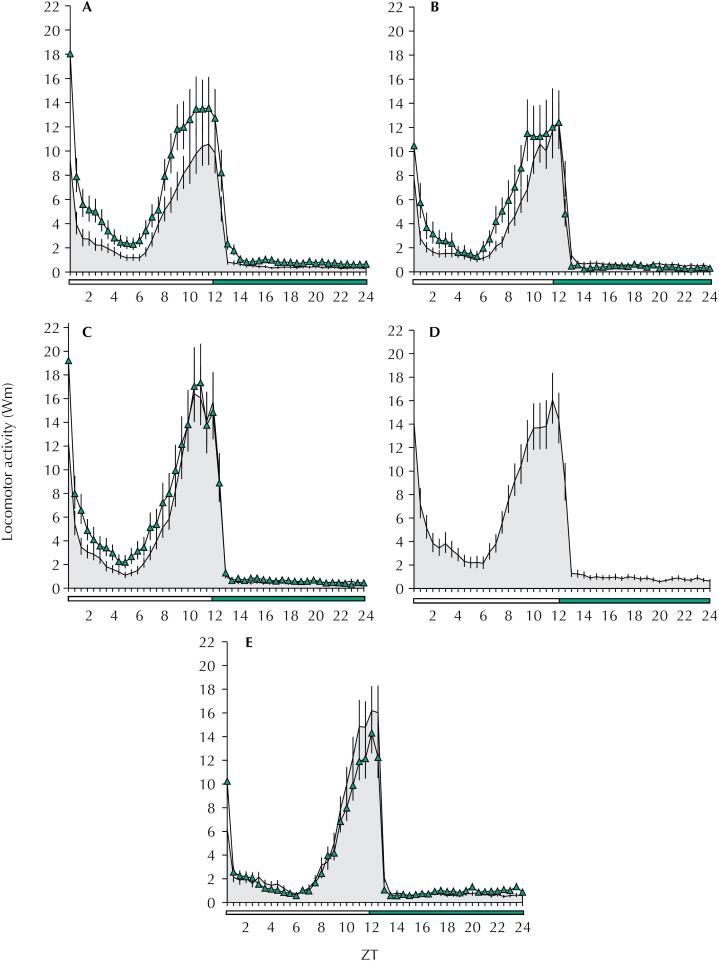
Locomotor activity of *Ae. aegypti* populations in 2016 and 2017. Locomotor activity of *Ae. aegypti* populations with reduced susceptibility (A – Campinas and B – Marília), resistant (C – Santos and D – Ribeirão Preto), and Rockefeller strain (E) in 2016 (grey area) and 2017 (line with triangles), under LD 12:12, at 25°C. Error bars are represented by solid (2016) and dashed (2017) lines. White bar represents the photophase and black bar the scothophase. ZT: *Zeitgeber* time; ZT0: time the light turns on; ZT12: time the light turns off.

Figure 2 shows locomotor activity of populations with reduced susceptibility, from Campinas (A) and Marília (B), resistant populations, from Santos (C) and Ribeirão Preto (only 2016) (D), and for the Rockefeller strain (E), all for 2016 and 2017, during the four days of experiment (days 2-5). All populations showed a diurnal and bimodal locomotor activity pattern, peaking at lights-on and near lights-off (Figure 2 A-E).

The 2017 populations from Campinas and Marília showed significantly increased locomotor activity during light phase when compared to 2016 populations (T-test, ZT06 to ZT12 interval; t = −2.991, p = 0.003 and t = −2.479, p = 0.014 respectively), suggesting a temporal variation (Figures 2A and B). However, we found no significant differences in the locomotor activity of Santos and Rockefeller populations between 2016 and 2017 (T-test, ZT06 to ZT12 interval; t = −1.555, p = 0.122 and t = 1.305, p = 0.194 respectively) (Figures 2C and E). As for Riberião Preto, no temporal comparison was possible as we only had data regarding 2016 (Figure 2D). The replicates of each population in 2016 and 2017 presented no significant difference (ANOVA, p > 0.05 and t-test, p > 0.05).

**Figure 3:**
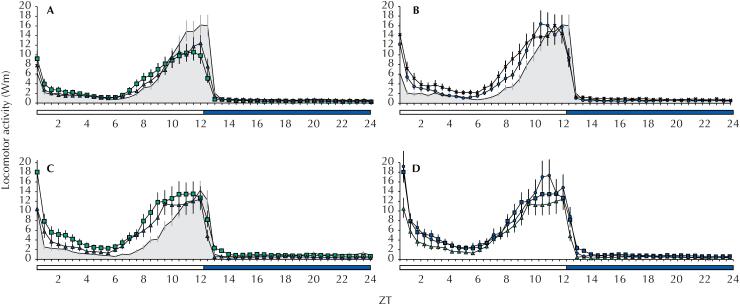
Locomotor activity of *Ae. aegypti* populations. 2016: (A) Populations with reduced susceptibility from Campinas (line with squares; error bars, solid line) and Marília (triangles; error bars, dark dashed line); (B) Resistant populations from Santos (circles; error bars, solid line) and Ribeirão Preto (crosses; error bars, dark dashed line). 2017: (C) Populations with reduced susceptibility from Campinas and Marília. (D) Resistant population from Santos and susceptible populations from Campinas and Marília. Locomotor activity of susceptible Rockefeller strain in 2016 and 2017 is represented in A, B, and C by a shaded grey area with dashed error bar. White bar represents the photophase and black bar the scothophase. ZT: *Zeitgeber* time; ZT0: time the light turns on; ZT12: time the light turns off.

Table 2 shows Fisher's LSD *post-hoc* results for *Ae. aegypti* populations with reduced susceptibility, resistant populations, and Rockefeller strain. In 2016, the Rockefeller strain showed a higher second peak compared to Marilia and Campinas (Figure 3A). However, both reduced susceptibility populations showed no significant difference in ZT06-ZT12 interval means when compared to the Rockefeller strain (p = 0.234 and p = 0.815, respectively) (Table 2). We also verified no significant differences between the populations of Marilia and Campinas (p = 0.296) (Table 2).

**Table 2:** Comparison between each population half-day activity (ZT06-ZT12) mean in relation to different insecticide resistance profiles.

Profile	Populations (I versus J)	2016		Populations (I versus J)	2017	
		Mean Difference (I-J)	p		Mean Difference (I-J)	p^a^
Reduced susceptibility/Susceptible	Camp *versus* Mar	0.040	0.296	Camp *versus* Mar	0.050	0.379
	Camp *versus* Rock	0.049	0.234	Camp *versus* Rock	0.232	**< 0.001**
	Mar *versus* Rock	0.009	0.815	Mar *versus* Rock	0.182	**0.003**
Reduced susceptibility/Resistant	Camp *versus* San	−0.085	**0.044**	Camp *versus* San	−0.038	0.446
	Camp *versus* Rib	−0.147	**< 0.001**	Mar *versus* San	−0.088	0.150
	Mar *versus* San	−0.125	**0.003**			
	Mar *versus* Rib	−0.186	**< 0.001**			
Susceptible/ Resistant	San *versus* Rock	0.135	**0.003**	San *versus* Rock	0.270	**< 0.001**
	Rib *versus* Rock	0.196	**< 0.001**			
Resistant	San *versus* Rib	−0.061	0.175			

Camp: Campinas; Mar: Marilia; San: Santos; Rib: Ribeirao Preto; Rock: Rockefeller.

a Results with ANOVA One-way and Fisher's LSD post hoc test.

Populations from Santos and Ribeirão Preto presented significantly increased locomotor activity when compared to the Rockefeller strain, especially during the anticipation period (from ZT06 to ZT12) (Figure 3B). ZT06-ZT12 interval means were significantly higher in resistant populations than in the reduced susceptibility populations from Campinas (p = 0.044 and p < 0.001, respectively) and Marília (p = 0.003 and p < 0.0001, respectively), and in the Rockefeller strain (p = 0.003 and p < 0.0001, respectively) (Table 2). However, we found no significant difference between the 2016 populations from Santos and Ribeirão Preto (p = 0.175) (Table 2).

Although 2017 populations from Campinas and Marília showed no significant differences in locomotor activity between them (p = 0.379) (Table 2), when compared to the Rockefeller strain locomotor activity was significantly increased (p < 0.0001 and p = 0.003, respectively), especially during the photophase (Figure 3C).

Contrary to 2016, Campinas and Marília populations did not differ significantly from Santos resistant population (p = 0.446 and p = 0.150, respectively) (Figure 3D; Table 2). Santos population differed significantly from the Rockefeller strain (p < 0.0001).

## DISCUSSION

*Ae. aegypti* resistant populations presented increased locomotor activity when compared to two populations with reduced susceptibility and to the susceptible Rockefeller laboratory strain. We also verified a temporal variation in the locomotor activity of both reduced susceptibility populations (Campinas and Marília): these populations showed lower locomotor activity than Santos and Ribeirão Preto resistant populations in 2016, but not in 2017. Chemical insecticides are still widely used to control *Ae. aegypti* mosquitoes, acting on their central nervous system, especially in axonal regions and at the voltage-gated sodium channel (NaV). Yet, the indiscriminate use of different insecticides classes has spurred random mutations, responsible for the development of insecticide resistance phenotype in different *Ae. aegypti* populations around the world^18^^,^^30^.

Brito et al. (2013) evaluated several life-cycle aspects in a resistant *Ae. aegypti* strain with *Kdr* mutations, using the Rockefeller strain as control group. According to the authors, when compared to the susceptible allele, the mutation probably has a deleterious maintenance cost or a higher fitness cost. In their study, the *Kdr* strain showed a significantly increased locomotor activity during the light phase when compared to the control group. Our results corroborate those reported by Brito et al. (2013), as *Ae. aegypti* populations from Ribeirão Preto and Santos, classified as resistant, showed more locomotor activity than populations with reduced susceptibility and the Rockefeller strain, especially in 2016^24^.

The 2016 populations from Campinas, Marília, and Rockefeller showed similar locomotor activity – significantly lower than that presented by the resistant populations from Santos and Ribeirão Preto. However, locomotor activity increased in Campinas and Marília in 2017, presenting significantly different values than the Rockefeller strain but not than Santos populations. This suggests a temporal variation that may be explained by changes in the pyrethroid mortality phenotype of these populations. In 2016, the mortality rate was 67% for the Campinas population and 96% for the Marília population; as for 2017, these values were 53% and 52%, respectively [Macoris MLG unpublished data]. Santos resistant population showed a lower mortality rate (28% in 2016 and 40% in 2017) than Campinas and Marília populations [Macoris MLG unpublished data]. This data could justify this population greater locomotor activity in relation to the Rockefeller strain in the two evaluated years, and in relation to populations with reduced susceptibility in 2016.

Previous studies have described temporal variation in resistance alleles frequency for all tested populations. In 2001, Santos showed a 0.24 frequency for the resistant allele 1016Ile^19^. Ten years later, resistant allele frequencies were 0.77 for Campinas, 0.53 for Marília, and 0.86, for Santos ^19^. In 2013, Ribeirão Preto presented a 0.68 frequency for the resistant allele 1016Ile, and 0.80 for 1534Cys [Nakazato BM and Bracco JE unpublished data]. In 2014, the last follow-up data, Campinas and Marília populations presented a 0.63 frequency for both 1016Ile and 1534Cys mutations. In the same year, both Santos and Ribeirão Preto populations also presented two fixed mutations (frequency 1.00)^15^. This finding may explain the similar locomotor activity results presented by Santos and Ribeirão Preto populations in 2016.

Few studies investigated the impact of insecticide resistance on *Ae. aegypti* locomotor activity, either in populations from São Paulo or elsewhere. Thus, our findings may help shedding light on several aspects directly involved in this mosquito's activity, such as blood-feeding, flight, and oviposition. We observed an increased locomotor activity of female *Ae. aegypti,* possibly contributing biologically to their broader dispersion, greater number of blood meals, or any other aspect affecting arboviruses transmission dynamics. A mosquito vectorial capacity is calculated considering several variables. Host-feeding, for examples, exert greater influence on a pathogen's basic reproductive rate (*R*0) than the abundance of mosquitoes^31^. Studies have suggested that the vector-biting rate is the most influential parameter for Zika virus transmission dynamics, causing the virus to invade a susceptible population^32^. DENV-2-infected female *Ae. aegypti* have been reported to present more locomotor activity than uninfected controls^22^. Such increased activity could raise infected mosquitoes biting rate, which, according to a mathematical model, could unfold into dengue outbreaks with greater numbers of primary and secondary infections and more severe biennial epidemics^23^. Thus, control measures should target reducing the vector-biting rate^32^. Mosquitoes circadian rhythms are mainly regulated by circadian clock neurons in the brain^33^, and mutations at sites in the mosquito central nervous system targeted by molecular pyrethroid are the major causes of resistance to this insecticide class^24^. Considering that, further studies should investigate whether these mutations are also associated with changes in locomotor activity.

Insecticide resistance in *Anopheles* mosquitoes, the main vectors of *Plasmodium* species, is a genuine concern throughout Africa, especially regarding *Anopheles gambiae*^34^. Different species of *Anopheles* mosquitoes resistant to insecticide also pose a concern for malaria control in other continents, including the Americas^35^. Few laboratory studies have compared the daily activities of resistant and susceptible *Anopheles* females^36^^-^^38^. Resistant *Anopheles gambiae* mosquitoes, for example, the main malaria vector in Africa, showed a shift in peak biting periods from 21:00-22:00hrs to 03:00-04:00hrs^36^. A study evaluated the flight activity of resistant and susceptible *Anopheles stephensi* females under laboratory conditions^37^, finding resistant females to show lower flight activity in the first two days than susceptible females^37^, differently than that found in our study. *Anopheles aquasalis* females fed with blood containing Ivermectin also showed a decrease in locomotor activity^38^. Further studies should be conducted on this important subject.

As insecticide-resistant populations pose a growing obstacle to vector control programs, our results may contribute to other studies aimed at elucidating aspects involving insecticide resistance, arbovirus infection, and locomotor activity in this mosquito vector.

## CONCLUSIONS

*Ae. aegypti* populations with insecticide resistance profile showed increased locomotor activity. In 2017, populations with reduced susceptibility presented an increase in the locomotor activity pattern, indicating a temporal change in relation to the previous year. The locomotor activity pattern in the populations from Campinas and Marília did not differ significantly from that found for Santos resistant population.

Our results indicate that insecticide-resistant *Ae. aegypti* populations show increased locomotor activity, which may affect their arboviruses transmission dynamics by increasing dispersion, number of blood meals, and other ecological parameters. Such results can shed light on these mechanisms action and effect in the context of an arbovirus epidemic, while helping to improve vector control strategies, innovations, and the epidemiology of this and other viral infections transmitted by *Ae. aegypti*.

## References

[1] 1. Braks MAH, Honório NA, Lourenço-de-Oliveira R, Juliano AS, Lounibos LP. Convergent habitat segregation of *Aedes aegypti* and *Aedes albopictus* (Diptera: Culicidae) in southeastern Brazil and Florida. J Med Entomol. 2003;40(6):785-94. https://doi.org/10.1603/0022-2585-40.6.785 10.1603/0022-2585-40.6.785 14765654

[2] 2. Lima-Camara TN. Activity patterns of *Aedes aegypti* and *Aedes albopictus* (Diptera: Culicidae) under natural and artificial conditions. Oecologia. 2010;14(3):737-44.

[3] 3. Lounibos LP, Kramer LD. Invasiveness of *Aedes aegypti* and *Aedes albopictus* and Vectorial capacity for Chikungunya Virus. J Infect Dis. 2016;214(Suppl 5):453-8. https://doi.org/10.1093/infdis/jiw285 10.1093/infdis/jiw285 PMC5137242 27920173

[4] 4. Brasil. Diretrizes nacionais para prevenção e controle de epidemias de dengue. 1st ver. ed. Brasília: Ministério da Saúde, 2009. 162 p.

[5] 5. Severo OP. Eradication of the *Aedes aegypti* mosquito from the Americas. Yellow fever, a symposium in commemoration of Carlos Juan Finlay; 1955; The Jefferson Medical College of Philadelphia, United States of America: Thomas Jefferson University; 1955. 20 p.

[6] 6. Dick OB, San Martín JL, Montoya RH, del Diego J, Zambrano B, Dayan GH. Review. The History of Dengue Outbreaks in the Americas. Am J Trop Med Hyg. 2012;87(4):584-93. https://doi.org/10.4269/ajtmh.2012.11-0770 10.4269/ajtmh.2012.11-0770 PMC3516305 23042846

[7] 7. Magalhães RCS. A erradicação do *Aedes aegypti*: febre amarela, Fred Soper e saúde pública nas Américas (1918-1968). Rio de Janeiro: Editora Fiocruz, 2016. 420 p. https://doi.org/10.7476/9788575414798

[8] 8. Braga IA, Valle D. *Aedes aegypti*: histórico de controle no Brasil. Epidemiol Serv Saúde. 2007;16(2):113-8. https://doi.org/10.5123/S1679-49742007000200006

[9] 9. Brasil. Portaria nº 329, de 02 de setembro de 1985. Diário Oficial da Únião. 1985 Set 2 [cited 2018 Apr 10]. Available from: http://bvsms.saude.gov.br/bvs/saudelegis/mapa_gm/1985/prt0329_02_09_198-5.html

[10] 10. Soderlund DM. Review Pyrethroids, knockdown resistance and sodium channels. Pest Manag Sci. 2008;64(6):610-6. 10.1002/ps.1574 18383430

[11] 11. D'amato C, Torres JPM, Malm O. DDT (Dicloro Difenil Tricloroetano); Toxicidade e Contaminação Ambiental - Uma Revisão. Quim Nova. 2002;25(6):995-1008. https://doi.org/10.1590/S0100-40422002000600017

[12] 12. Superintendência de Controle de Endemias. Suplemento Especial do Boletim Epidemiológico Paulista. São Paulo: Secretaria da Saúde do Governo do Estado de São Paulo, 2006. 62 p.

[13] 13. Macoris MLG, Andrighetti MTM, Wanderley DMV, Ribolla PEM. Impact of insecticide resistance on the field control of *Aedes aegypti* in the State of São Paulo. Rev Soc Bras Med Trop. 2014;47(5):573-8. https://doi.org/10.1590/0037-8682-0141-2014 10.1590/0037-8682-0141-2014 25467257

[14] 14. Macoris MLG, Martins AJ, Andrighetti MTM, Lima JBP, Valle D. Pyrethroid resistance persists after ten years without usage against *Aedes aegypti* in governmental campaigns: Lessons from São Paulo State, Brazil. PLoS Negl Trop Dis. 2018;12(3):1-18. https://doi.org/10.1371/journal.pntd.0006390 10.1371/journal.pntd.0006390 PMC5895049 29601580

[15] 15. World Health Organization. Monitoring and managing insecticide resistance in *Aedes* mosquito populations: interim guidance for entomologists. Geneva: World Health Organization, 2016. 13 p.

[16] 16. Hemingway J, Hanson H. Insecticide Resistance in Insect Vectors of Human Disease. Annu Rev Entomol. 2000;45:371-91. https://doi.org/10.1146/annurev.ento.45.1.371 10.1146/annurev.ento.45.1.371 10761582

[17] 17. Batista E. Evolução de mutações do gene do canal de sódio associadas à resistência tipo Kdr em populações de *Aedes (Stegomyia) aegypti* do Estado de São Paulo [master's dissertation]. São Paulo: School of Public Health, University of São Paulo, 2012.

[18] 18. Martins AJ, Lima JBP, Peixoto AA, Valle D. Frequency of Val1016Ile mutation in the voltage-gated sodium channel gene of *Aedes aegypti* Brazilian populations. Trop Med Int Health. 2000;14(11):1351-5. https://doi.org/10.1111/j.1365-3156.2009.02378.x 10.1111/j.1365-3156.2009.02378.x 19735371

[19] 19. Linss JGB, Brito LP, Garcia GA, Araki AS, Bruno RV, Lima JBP, et al. Distribution and dissemination of the Val1016Ile and Phe1534Cys Kdr mutations in *Aedes aegypti* Brazilian natural populations. Parasit Vectors. 2014;7(25):1-11. https://doi.org/10.1186/1756-3305-7-25 10.1186/1756-3305-7-25 PMC3912884 24428880

[20] 20. Saunders DS. Insect Clocks, Third edition. Elsevier Science. University of Edinburgh. 1st rev. ed. Amsterdam: Elsevier, 2002. 576 p.

[21] 21. Lima-Camara TN, Lima JBP, Bruno RV, Peixoto AA. Effects of insemination and blood-feeding on locomotor activity of *Aedes albopictus* and *Aedes aegypti* (Diptera: Culicidae) females under laboratory conditions. Parasit Vectors. 2014;7(304):1-8. https://doi.org/10.1186/1756-3305-7-304 10.1186/1756-3305-7-304 PMC4094679 24990394

[22] 22. Lima-Camara TN, Bruno RV, Luz PM, Castro MG, Lourenço-de-Oliveira R, Sorgine MHF, et al. Dengue Infection Increases the Locomotor Activity of *Aedes aegypti* Females. PLoS One. 2011;6(3):e17690. https://doi.org/10.1371/journal.pone.0017690 10.1371/journal.pone.0017690 PMC3050906 21408119

[23] 23. Luz PM, Lima-Camara TN, Bruno RV, Castro MG, Sorgine, MHF, Lourenço-de-Oliveira R, et al. Potential impact of a presumed increase in the biting activity of dengue-virus-infected *Aedes aegypti* (Diptera: Culicidae) females on virus transmission dynamics. Mem Inst Oswaldo Cruz. 2011;106(6):755-8. https://doi.org/10.1590/S0074-02762011000600017 10.1590/s0074-02762011000600017 22012232

[24] 24. Brito LP, Linss JGB, Lima-Camara TN, Belinato TA, Peixoto AA, Lima JBP, et al. Assessing the Effects of *Aedes aegypti* kdr Mutations on Pyrethroid Resistance and Its Fitness Cost. PLoS One. 2013;8(4):e60878. https://doi.org/10.1371/journal.pone.0060878 10.1371/journal.pone.0060878 PMC3620451 23593337

[25] 25. Rosato E, Kyriacou CP. Analysis of locomotor activity rhythms in *Drosophila*. Nat Protoc. 2006;1(2):559-68. https://doi.org/10.1038/nprot.2006.79 10.1038/nprot.2006.79 17406282

[26] 26. Moore-Ede M, Sulzman FM, Fuller CA. The clocks that time us: physiology of the circadian timing system. Cambridge: Harvard University Press, 1984. https://doi.org/10.1152/ajpregu.1986.250.5.R737

[27] 27. Lima-Camara TN, Codeço CT, Honório NA, Bruno RV, Peixoto AA, Lounibos LP. Male accessory gland substances from *Aedes albopictus* affect the locomotor activity of *Aedes aegypti* females. Mem Inst Oswaldo Cruz. 2013;108(Suppl. 1):18-25. https://doi.org/10.1590/0074-0276130381 10.1590/0074-0276130381 PMC4109176 24473799

[28] 28. Alexander N. Review: analysis of parasite and other skewed counts. Trop Med Int Health. 2012;17(6): 684-93. https://doi.org/10.1111/j.1365-3156.2012.02987.x 10.1111/j.1365-3156.2012.02987.x PMC3468795 22943299

[29] 29. Taylor B, Jones MD. The circadian rhythm of flight activity in the mosquito *Aedes aegypti* (L.): the phase-setting effects of light-on and light-off. J Exp Biol. 1969;51(1):59-70. 10.1242/jeb.51.1.59 5387705

[30] 30. Braga IA, Valle D. *Aedes aegypti*: Insecticides, Mechanisms of Action and Resistance. Epidemiol Serv Saúde. 2007;16(4):279-93. https://doi.org/10.5123/S1679-49742007000400006

[31] 31. Lounibos LP, Kramer LD. Invasiveness of *Aedes aegypti* and *Aedes albopictus* and Vectorial Capacity for Chikungunya Virus. J Infect Dis. 201615;214(Suppl 5):453-458. https://doi.org/10.1093/infdis/jiw285 10.1093/infdis/jiw285 PMC5137242 27920173

[32] 32. Wachira CM, Lawi OG, and Malinzi J. A spatiotemporal model on the transmission dynamics of Zika virus disease. Asian J Math. 2018;10(4):1-15. https://doi.org/10.9734/ARJOM/2018/43944

[33] 33. Meirelles-Filho ACA, Kyriacou CP. Circadian rhythms in insect disease vectors. Mem Inst Oswaldo Cruz. 2013;108(Suppl. 1):48-58. https://doi.org/10.1590/0074-0276130438 10.1590/0074-0276130438 PMC4109179 24473802

[34] 34. Ranson H, Lissenden N. Insecticide Resistance in African *Anopheles* Mosquitoes: A Worsening Situation that Needs Urgent Action to Maintain Malaria Control. Trends Parasitol. 2016;32(3):187-196. https://doi.org/10.1016/j.pt.2015.11.010 10.1016/j.pt.2015.11.010 26826784

[35] 35. Silva GL, Pereira TN, Ferla NJ, Silva OS. The impact of insecticides management linked with resistance expression in *Anopheles* spp. populations. Cienc Saude Colet. 2016;21(7):2179-88. https://doi.org/10.1590/1413-81232015217.00922015 10.1590/1413-81232015217.00922015 27383351

[36] 36. Githinji EK, Irungu LW, Ndegwa PN, Machani MG, Amito RO, Kemei BJ, et al. Impact of Insecticide Resistance on *P. falciparum* Vectors’ Biting, Feeding, and Resting Behaviour in Selected Clusters in Teso North and South Subcounties in Busia County, Western Kenya. J Parasitol Res. 2020;2020:9423682. https://doi.org/10.1155/2020/9423682 10.1155/2020/9423682 PMC7168709 32328298

[37] 37. Rowland M. Activity and mating competitiveness of γHCH/dieldrin resistant and susceptible male and virgin female *Anopheles gambiae* and *An.stephensi* mosquitoes, with assessment of an insecticide‐rotation strategy. Med Vet Entomol. 1991;5(2): 207-22.https://doi.org/10.1111/j.1365-2915.1991.tb00543.x 10.1111/j.1365-2915.1991.tb00543.x 1722729

[38] 38. Sampaio VS, Rivas GBS, Kobylinski K, Pinilla YT, Pimenta PFP, Lima JBP, et al. What does not kill it makes it weaker: effects of sub-lethal concentrations of ivermectin on the locomotor activity of *Anopheles aquasalis*. Parasit Vectors. 2017;10(10):623. https://doi.org/10.1186/s13071-017-2563-0 10.1186/s13071-017-2563-0 PMC5745606 29282130

